# Development and validation of novel risk prediction models of breast cancer based on stanniocalcin‐1 level

**DOI:** 10.1002/cam4.5419

**Published:** 2022-11-06

**Authors:** Sheng Huang, Yuyuan Chen, Jiong Wu, Yayun Chi

**Affiliations:** ^1^ Department of Breast Surgery, Breast Cancer Institute Fudan University Shanghai Cancer Center, Fudan University Shanghai China; ^2^ The 2nd Department of Breast Surgery The Third Affiliated Hospital of Kunming Medical University Kunming China; ^3^ The Department of Thyroid and Breast Surgery The Affiliated Hospital of Ningbo University Medical College Ningbo China

**Keywords:** breast cancer, disease‐free survival, distant disease‐free survival, overall survival, Stanniocalcin‐1

## Abstract

**Purpose:**

The function of stanniocalcin‐1 (STC‐1) in the oncogenesis and progression of tumors has been extensively studied. The purpose of this study was to investigate the relationship between secreted STC‐1 and prognosis in patients with breast cancer (BC) and to determine whether STC‐1 could be a key prognostic factor in BC.

**Methods:**

The STC‐1 level was measured by ELISA and clinical data from 1210 female patients with BC were used to develop and validate nomograms. We then verified the models through the plotting of ROC curves and calibration curves, calculating the C‐index, and performing decision curve analyses (DCA).

**Results:**

The level of STC‐1 in the peripheral plasma was significantly correlated with the T stage, N stage, clinical stage, grade, hormone receptors, HER‐2 status, and tumor subtype. Cox regression analyses revealed that estrogen receptor(ER) status, N stage, and STC‐1 level were risk factors for overall survival (OS), whereas T stage, N stage, and STC‐1 level were independent prognostic factors for distant disease‐free survival (DDFS) and disease‐free survival (DFS). Both the ROC curve and the C‐index confirmed the high resolution of these models, while the DCA identified the feasibility of their practical application. In addition, the calibration curves indicated good consistency between the predicted and actual survival rates.

**Conclusion:**

Nomograms were created based on STC‐1 levels for 3‐, 5‐, and 7‐year OS, DDFS, and DFS of patients with BC respectively. As a key prognostic factor for BC, peripheral blood STC‐1 level can be used clinically as a liquid biopsy indicator.

## Graphical abstract

In this study, we used stanniocalcin‐1 levels in the peripheral blood of patients with breast cancer as an independent prognostic factor and constructed nomograms with clinicopathological information. Prediction models based on STC‐1 level for prediction of OS, DDFS, and DFS at 3, 5, and 7 years have good predictive ability for BC and have potential clinical value.

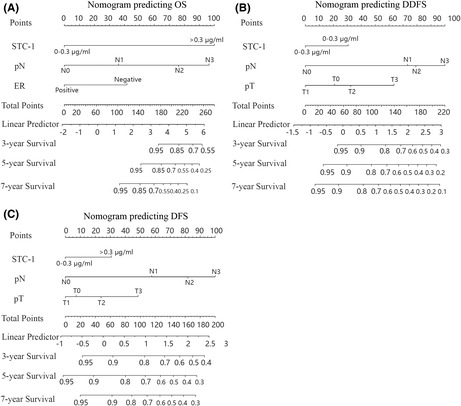

## 1 INTRODUCTION

As one of the most prevalent malignant tumors, breast cancer (BC) severely affects people's lives and health.^1^ The etiology and pathogenesis of BC are complex and highly heterogeneous.^2^, ^3^ With the emergence of more targeted drugs, the treatment of BC and the prognosis of patients have been greatly improved.

Stanniocalcin (STC)‐1 is a glycoprotein that was first identified in the stannius bodies of fish.^4^, ^5^, ^6^ STC‐1, a member of the STC family, participates in regulating serum calcium and phosphate metabolism.^7^, ^8^ Autocrine or paracrine mechanisms are involved in secreting STC‐1 into extracellular matrix.^6^, ^9^ STC‐1 has been reported to contribute to cancer progression by inhibiting apoptosis; promoting the vitality, proliferation, and invasiveness of cancer cells; and promoting tumor chemical resistance.^8^, ^10^

According to multiple studies, the high expression of STC‐1 in BC is often linked with poor prognoses.^10^, ^11^, ^12^, ^13^, ^14^ Joensuu et al. performed an immunohistochemical analysis of 72 primary BC tissue samples and found that the expression levels of STC‐1 and STC‐2 in metastases at 5 and 10 years after surgery were significantly higher than those in early metastases.^15^ In our previous study, we revealed that STC‐1 promotes metastasis in BC and that STC‐1 transcription is activated by CAPG.^16^ Therefore, we investigated whether the prognosis of patients can be predicted by measuring the level of secreted STC‐1 in the patient's blood.

Nomograms are based on several risk factors that can be commonly applied to predict disease outcomes.^17^, ^18^, ^19^, ^20^ It remains unclear how secreted STC‐1 can be used to predict survival prognosis in BC. The purpose of this research was to develop prognostic nomograms with clinical value by combining STC‐1 levels in peripheral blood with clinical and pathological factors in patients with BC.

## 2 PATIENTS AND METHODS

### 2.1 Patient sample and ELISA

Peripheral plasma of patients with BC was obtained from the Breast Surgery Department at Fudan University Shanghai Cancer Center (FDUSCC, Shanghai, PR China). Information about clinicopathological features and follow‐up of the patients were collected according to previous methods.^21^ The Ethics Committee of the FDUSCC approved this research, and each patient provided informed consent. An enzyme‐linked immunosorbent assay (ELISA) (Human Stanniocalcin 1 DuoSet ELISA, DY2958, R&D Systems) was performed to determine STC‐1 in peripheral plasma and assign STC‐1 values to patients with BC using STC‐1 levels in normal subjects as a reference. The optimal cut‐off value for STC‐1 was 0.3 μg/ml using X‐tile software. Correlations between STC‐1 expression and clinicopathological information were analyzed by the Wilcoxon test between two groups and the Kruskal‐Wallis test for analysis between multiple groups.

### 2.2 Variable declaration

The patients were screened in accordance with the following inclusion criteria: female sex, absence of metastases, known age, family history of BC, breast subtype, and stage (Figure S1). We screened 1210 patients and divided them into a training group and a validation group. The median age of the included population was taken as a cut‐off to classify patients into younger and older groups.

Patient characteristics included age, family history, histological type, stage, grade, hormone receptors (HR), human epidermal growth factor receptor‐2(HER‐2) status, Ki‐67 status, and vascular tumor emboli.

### 2.3 Statistical analysis

Univariate Cox analysis was conducted based on OS, DDFS, and DFS using the SPSS software (version 25.0). R version 4.1.1 was used to load various R packages and codes. The respective nomograms of OS, DDFS, and DFS were constructed by “rms” package. The relationship between risk factors and prognosis was verified by plotting Kaplan–Meier survival curves combined with log‐rank tests.

A concordance index (C‐index) above 0.70 shows that the model has good accuracy. The C‐index of each dataset was calculated by “survival” package and “coxph” function. The factors included in the nomograms were integrated, calculated, and assigned risk values, and a truncated median value was used to divide the entire dataset into high‐ and low‐risk groups. ROC curves for OS, DDFS, and DFS were plotted for 3, 5, and 7 years by “survivalROC” package. Calibration curves and DCA curves were plotted to determine the accuracy of the nomograms and clinical application scopes.

## 3 RESULTS

### 3.1 Expression of STC‐1

**FIGURE 1:**
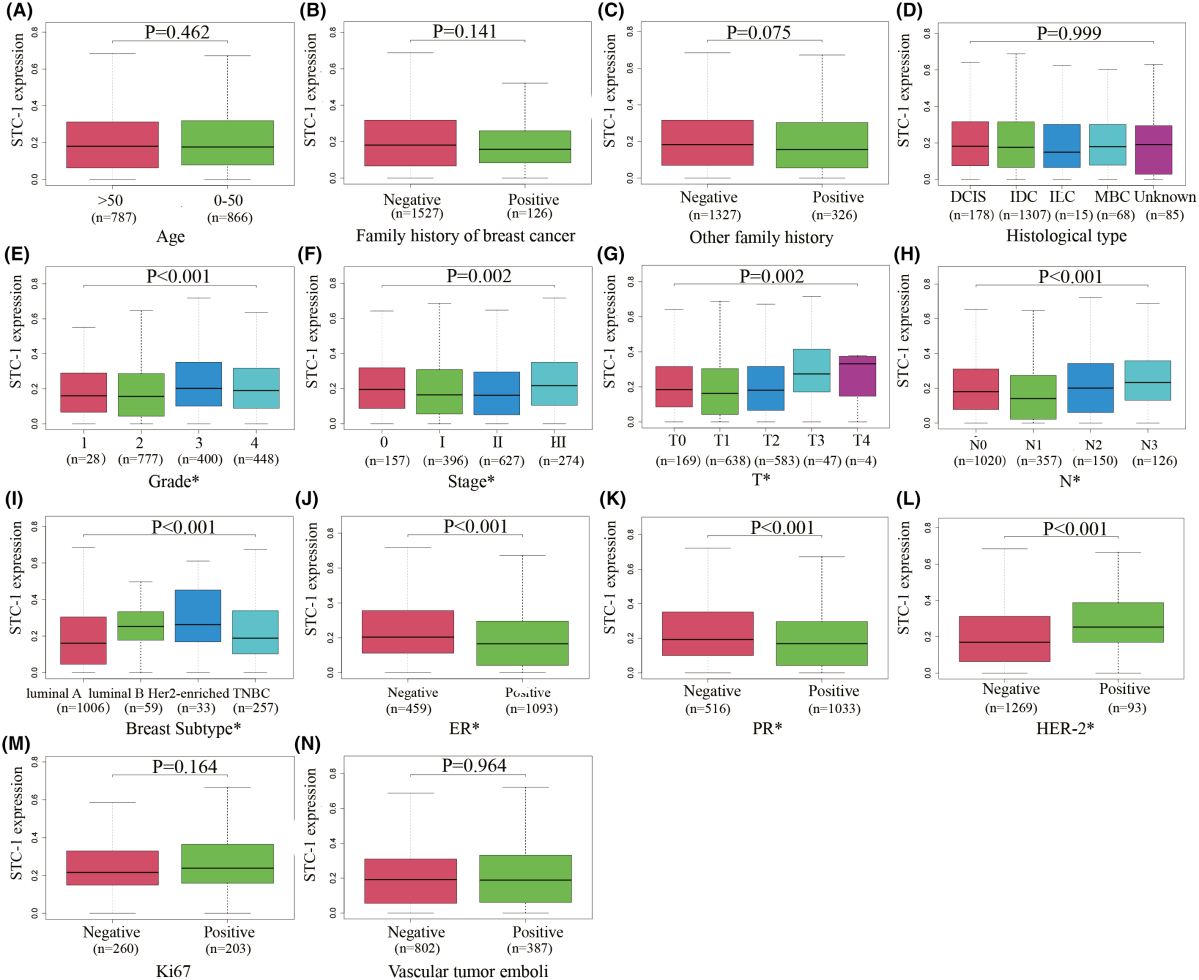
Relationships between STC‐1 expression and clinicopathological features of BC (*p < 0.05). (A) Age; (B) Family history of breast cancer; (C) Other family history; (D) Histological type; (E) Grade; (F) Stage; (G) T stage; (H) N stage; (I) Breast subtype; (J) ER status; (K) PR status; (L) HER‐2 status; (M) Ki67 status; (N) Vascular tumor emboli.

There were 1653 peripheral blood samples were collected from the FDUSCC. Correlation analyses between STC‐1 and clinical characteristics revealed that the difference in STC‐1 expression levels was significantly associated with grade (*p* < 0.001), American Joint Committee on Cancer (AJCC) stage (*p* = 0.002), T stage (*p* = 0.002), N stage (*p* < 0.001), breast subtype (*p* < 0.001), estrogen receptor(ER) status (*p* < 0.001), progesterone receptor(PR) status (*p* < 0.001), HER‐2 status (*p* < 0.001), but was not associated with age, family history, histological type, Ki67, or vascular tumor emboli (*p* > 0.05) (Figure 1). STC‐1 expression was higher in ER‐positive, PR‐negative, or HER‐2‐positive BC samples.

### 3.2 Clinicopathological characteristics of the included patients

After screening, information on 1210 patients with BC was included in the model development and validation. The age of the entire population was 51 ± 11.65 years and 51 years was used as the cut‐off value. Seventy percent of the patients were randomized to the training group, while the remaining 30% were classified as the validation group. As is shown in Table 1, 79.2% had a family history of cancer, of which 92.3% had a family history of BC. Among the patients, 7.5%, 28.5%, 45.3%, and 18.6% were in stages 0, I, II, and III, respectively. The majority of breast subtypes were luminal A, and only 2.5% were HER‐2 enriched. Tumor grade was I in 1.5% of the patients, II in 52.8%, III in 27.4%, and IV in 18.3%. Of the vascular tumor emboli, 54.2% were negative, 26.1% were positive, and the remainder were unknown. Ki67 information was lacking in 67%. Patients with low STC‐1 expression and those with high STC‐1 expression were in the proportion of 72.7% and 27.3%, respectively.

**TABLE 1:** Characteristics of BC patients

Variables	Total cohort		Training cohort		Validation cohort	
	*N* = 1210		*N* = 849		*N* = 361	
	*n*	%	*N*	%	*n*	%
Age						
<51	580	47.9	404	47.6	176	48.8
≥51	630	52.1	445	52.4	185	51.2
Family history of BC						
No	1118	92.4	784	92.3	334	92.5
Yes	92	7.6	65	7.7	27	7.5
Other family history						
No	963	79.6	672	79.2	291	80.6
Yes	247	20.4	177	20.8	70	19.4
Stage						
0	98	8.1	64	7.5	34	9.4
I	348	28.8	242	28.5	106	29.4
II	541	44.7	385	45.3	156	43.0
III	223	18.4	158	18.6	65	18.0
T						
T0	106	8.8	67	7.9	39	10.8
T1	560	46.3	393	46.3	167	46.3
T2	503	41.6	366	43.1	137	38.0
T3	41	3.4	23	2.7	18	5.0
*N*						
N0	704	58.2	494	58.2	210	58.2
N1	290	24.0	201	23.7	89	24.7
N2	114	9.4	86	10.1	28	7.8
N3	102	8.4	68	8.0	34	9.4
Grade						
I	22	1.8	13	1.5	9	2.5
II	639	52.8	448	52.8	191	52.9
III	326	26.9	233	27.4	93	25.8
IV	223	18.4	155	18.3	68	18.8
Breast subtype						
HR+/HER2‐ (Luminal A)	899	74.3	625	73.6	274	75.9
HR+/HER2+ (Luminal B)	57	4.7	41	4.8	16	4.4
HR‐/HER2+ (HER2 enriched)	28	2.3	21	2.5	7	1.9
HR‐/HER2‐ (Triple Negative)	226	18.7	162	19.1	64	17.7
ER						
Negative	307	25.4	223	26.3	84	23.3
Positive	903	74.6	626	73.7	277	76.7
PR						
Negative	371	30.7	261	30.7	110	30.5
Positive	839	69.3	588	69.3	251	69.5
HER‐2						
Negative	1125	93.0	787	92.7	338	93.6
Positive	85	7.0	62	7.3	23	6.4
Ki67						
Negative	206	17.0	151	17.8	55	15.2
Positive	192	15.9	125	14.7	67	18.6
Unknown	812	67.1	573	67.5	239	66.2
Histological type						
DCIS	112	9.3	69	8.1	43	11.9
IDC	995	82.2	703	82.8	292	80.9
ILC	12	1.0	9	1.1	3	0.8
MBC	46	3.8	32	3.8	14	3.9
Unknown	45	3.7	36	4.2	9	2.5
Vascular tumor emboli						
Negative	648	53.6	460	54.2	188	52.1
Positive	324	26.8	222	26.1	102	28.3
Unknown	238	19.7	167	19.7	71	19.7
STC‐1						
0–0.3 μg/ml	877	72.5	617	72.7	260	72.0
>0.3 μg/ml	333	27.5	232	27.3	101	28.0

Abbreviations: DCIS, Ductal carcinoma in situ; IDC, Invasive ductal carcinoma; ILC, Invasive lobular carcinoma; MBC, Mucinous breast carcinoma.

### 3.3 Prognostic factors

**FIGURE 2:**
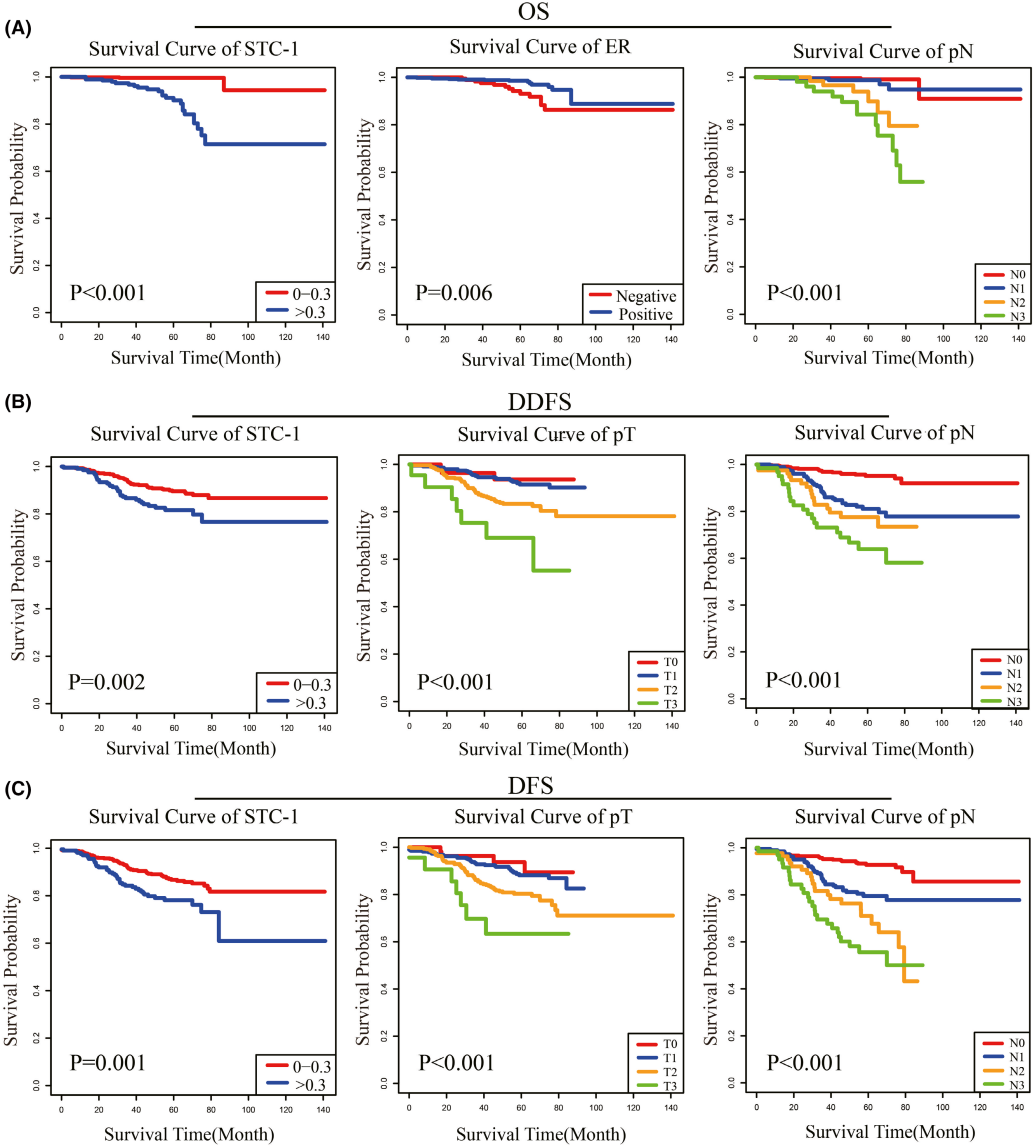
Kaplan–Meier curves of prognostic factors in patients with BC. (A) Risk factors for predicting OS: STC‐1, ER status, and N stage. (B) Risk factors for predicting DDFS: STC‐1, T stage, and N stage. (C) Risk factors for predicting DFS: STC‐1, T stage, and N stage.

Cox analyses were performed on OS, DDFS, and DFS of the training set. In the OS‐based univariate analysis, T stage, N stage, ER status vascular tumor emboli, and STC‐1 expression were statistically significant. To avoid multicollinearity, the clinical stage, T stage, and N stage could not be included in the multifactorial analysis simultaneously. N2 stage, N3 stage, and STC‐1 > 0.3 μg/ml were considered risk factors for OS of BC by multifactorial analysis, while ER‐positive status was a protective factor (Table S1). The same methods were used to calculate Cox regression analyses based on DDFS and DFS. In the results of the DDFS‐based multivariate analysis, T stage, N stage, and higher expression of STC‐1 were independent prognostic factors (Table S2). In addition, N stage and STC‐1 > 0.3 μg/ml were also identified to be significantly associated with DFS in BC (Table S3). Moreover, survival curves were plotted for each independent risk factor to determine the effect on BC prognosis, and all *p* values were statistically significant by log‐rank tests (Figure 2).

**FIGURE 3:**
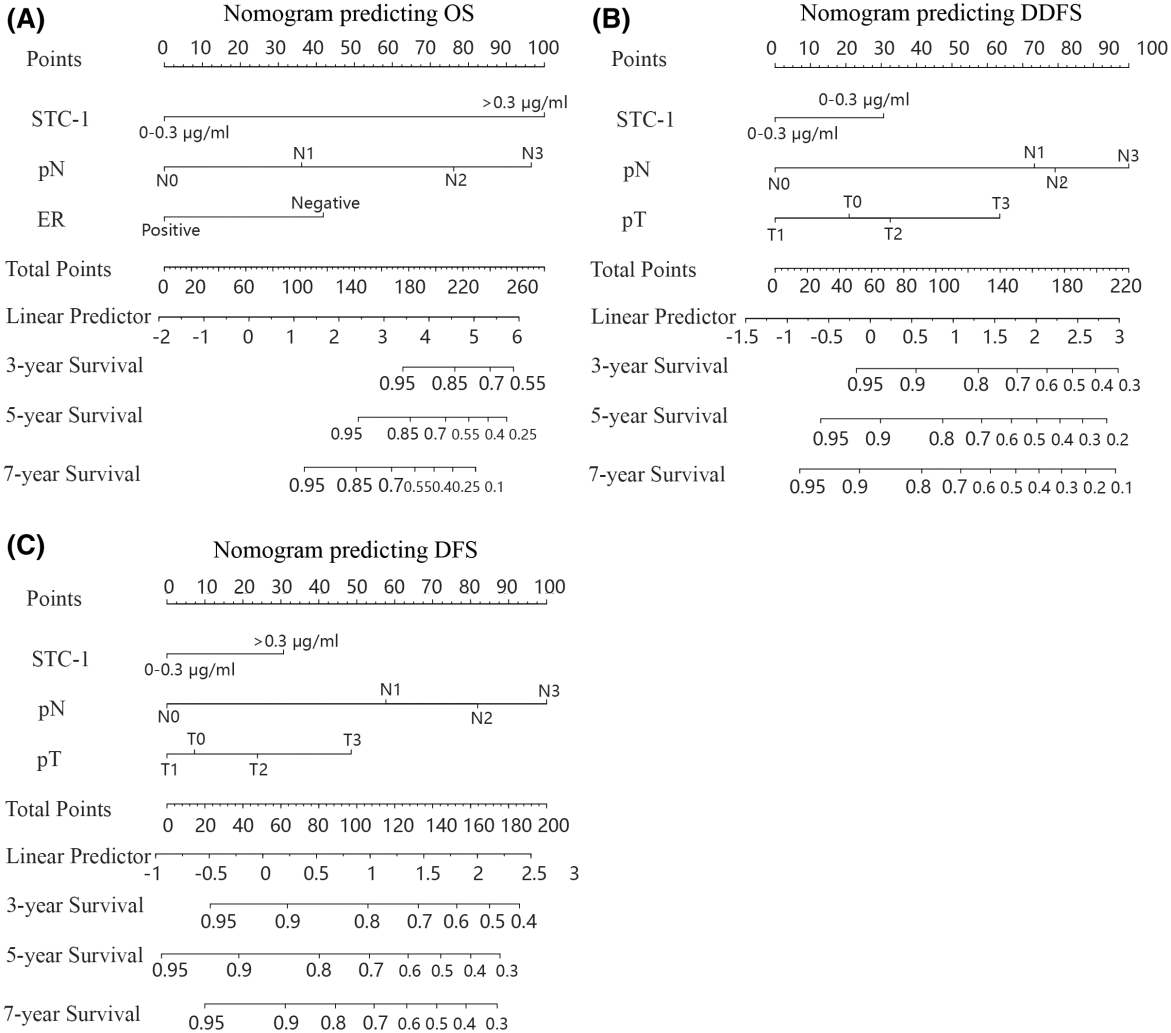
Nomograms predicting OS, DDFS, and DFS in patients with BC. (A) 3, 5, 7 years OS. (B) 3, 5, 7 years DDFS. (C) 3, 5, 7 years DFS.

The identified independent risk factors were used to construct prognostic nomograms to predict 3‐, 5‐, and 7‐year OS, DDFS, and DFS (Figure 3). Although the *p*‐value for the T stage in the multifactorial analysis of DFS was >0.05, given the importance of the T stage in the clinical prediction model, it was also included in the nomogram. The nomogram predicting OS revealed that STC‐1 was the primary risk factor, followed by N stage and ER status, while N stage was the most critical factor affecting prognosis for both DDFS and DFS, followed by T stage and STC‐1.

### 3.4 Validation of nomograms

**FIGURE 4:**
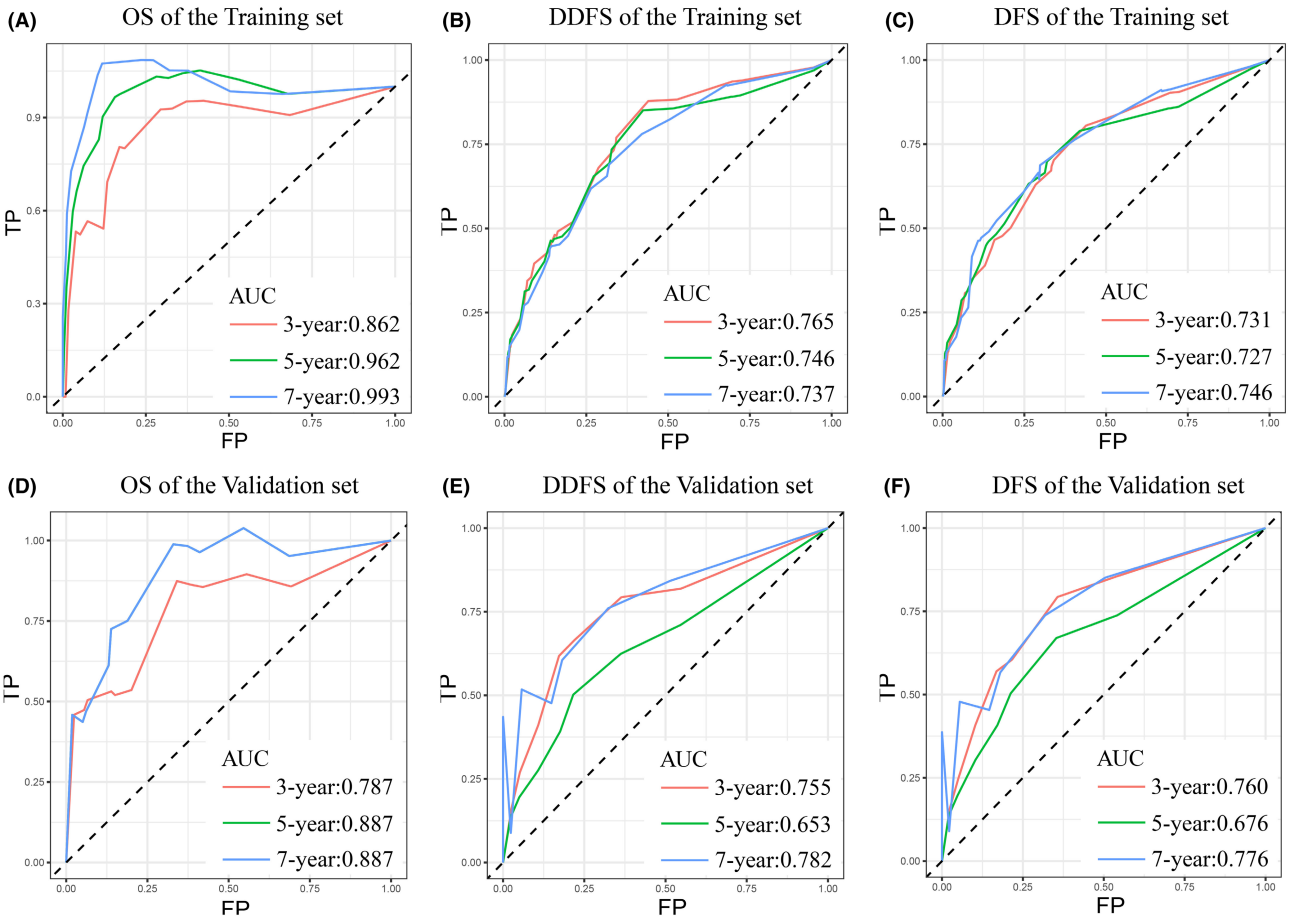
ROC curve with the area under the curve (AUC) values for OS, DDFS, and DFS in patients with BC. (A) 3, 5, 7 years OS rate in the training set. (B) 3, 5, 7 years DDFS rate in the training set. (C) 3, 5, 7 years DFS rate in the training set. (D) 3, 5, 7 years OS rate in the validation set. (E) 3, 5, 7 years DDFS rate in the validation set. (F) 3, 5, 7 years DFS rate in the validation set.

**FIGURE 5:**
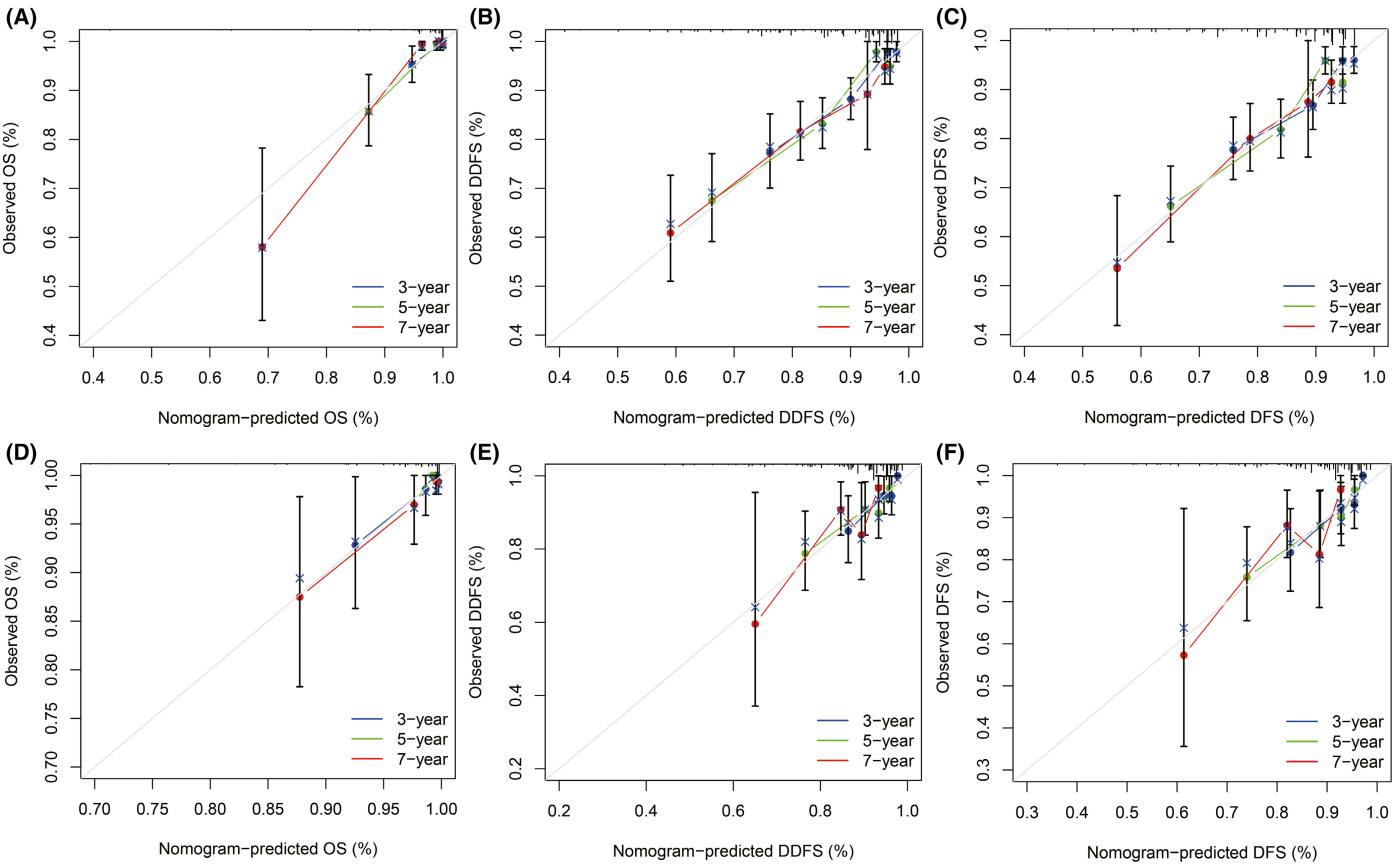
The 3, 5, 7 years calibration curves of nomograms for predicting OS, DDFS, and DFS. (A) OS in the training set. (B) DDFS in the training set. (C) DFS in the training set. (D) OS in the validation set. (E) DDFS in the validation set. (F) DFS in the validation set.

The accuracy and precision of nomograms are critical for the practical application of prognostic models. The C‐index estimates the probability that the predicted outcome will be consistent with the actual observed outcome. It is commonly used to evaluate the predictive power of the model. For the validation of the predicted OS, DDFS, and DFS nomograms, the C‐indexes were 0.888, 0.746, and 0.711 for the training set and 0.807, 0.697, 0.705 for the validation set, respectively (Table S4). Time‐dependent ROC curves can reflect the predictive effect of one indicator on the outcome at different time points. The larger the area under the ROC curve, the better the differentiation ability of the model. In the training cohort, the AUCs of the models for 3‐, 5‐, and 7‐year OS were predicted to be 0.862, 0.962 and 0.993, 0.765, 0.746, and 0.737 for DDFS and 0.731, 0.727, and 0.746 for DFS, respectively (Figure 4A–C). In the validation cohort, the AUCs of the models for 3‐, 5‐, and 7‐year OS were predicted to be 0.787, 0.887 and 0.887, 0.755, 0.653, and 0.782 for DDFS and 0.760, 0.676, and 0.776 for DFS, respectively (Figure 4D–F). The calibration curves plotted based on OS, DDFS, and DFS also showed good consistency between the nomogram predictions and actual survival (Figure 5A–F).

**FIGURE 6:**
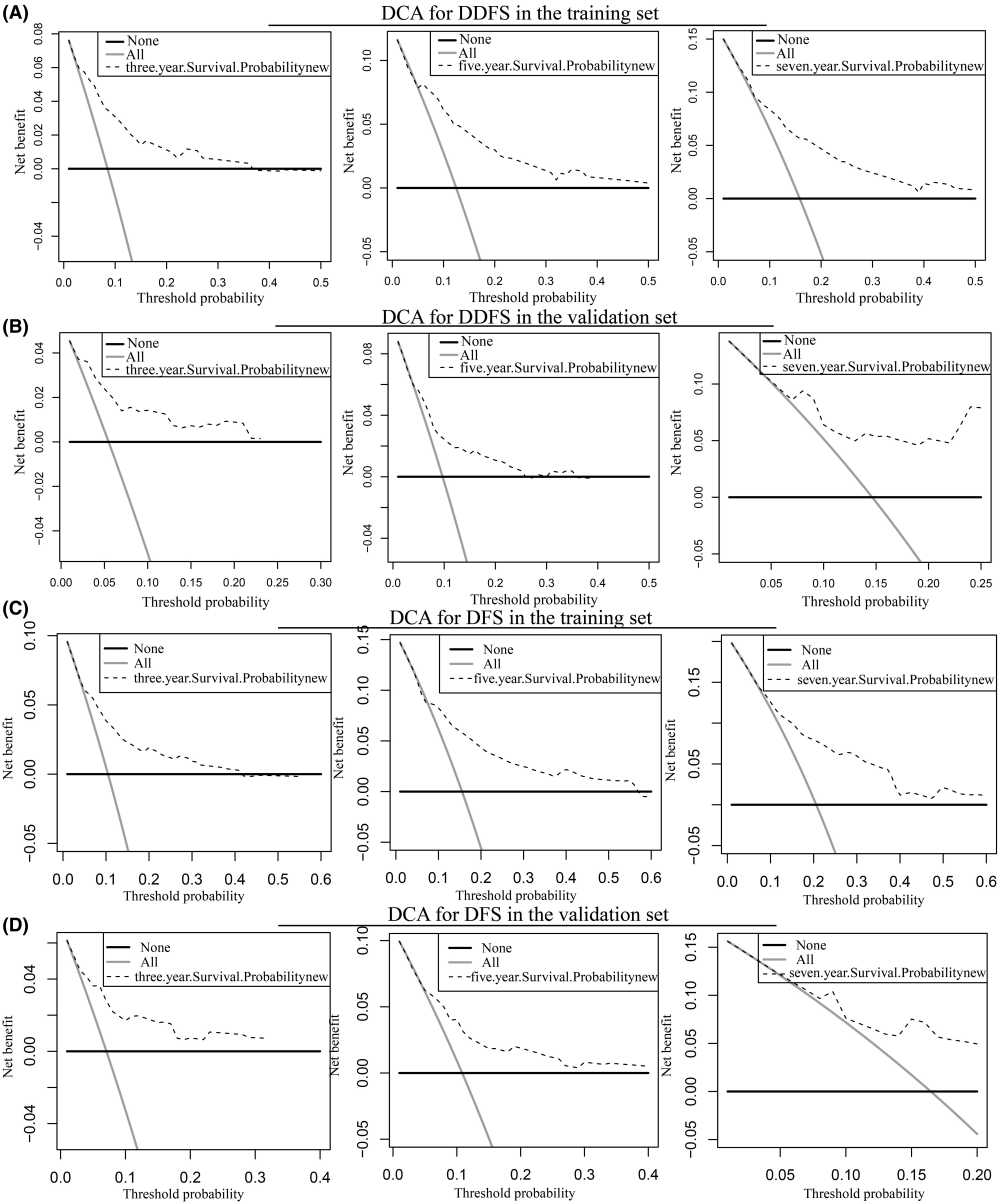
Decision curve analyses (DCA) of nomograms for predicting DDFS and DFS. (A) 3, 5, 7 years DCA for predicting DDFS in the training set. (B) 3, 5, 7 years DCA for predicting DDFS the validation set. (C) 3, 5, 7 years DCA for predicting DFS in the training set. (D) 3, 5, 7 years DCA for predicting DFS the validation set.

**FIGURE 7:**
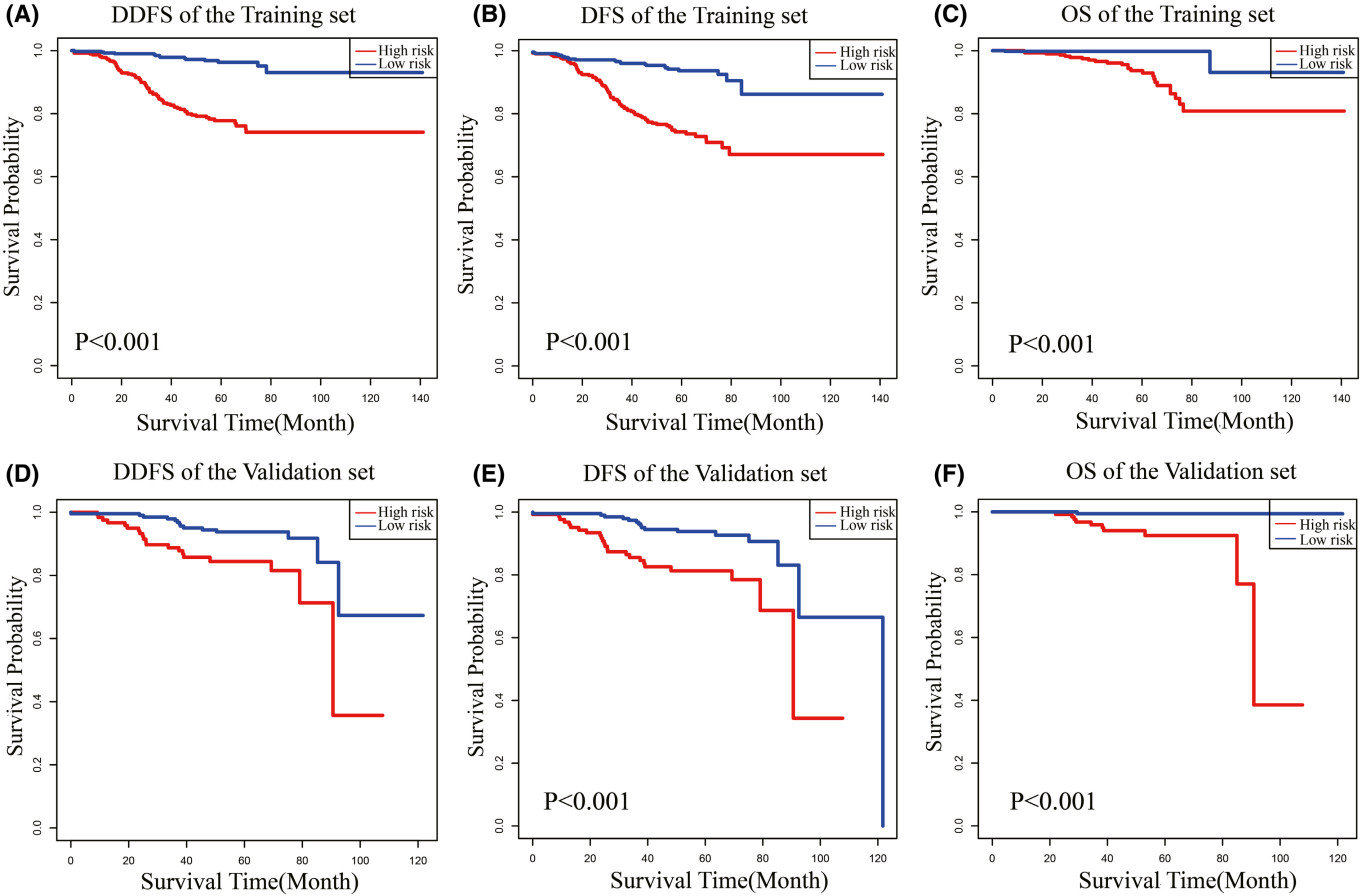
Kaplan–Meier survival curves for OS, DDFS, and DFS of patients with BC in different risk subgroups. (A–C) The training set predicts the survival curve of OS, DDFS, and DFS. (D–F) The validation set predicts the survival curve of OS, DDFS, and DFS.

The net benefit helps determine whether the benefits of a clinical decision based on a model, marker, or test outweigh the disadvantages.^22^ The decision curve analysis (DCA) can determine the clinical usefulness of nomograms by quantifying the net benefit at different threshold probabilities.^22^, ^23^, ^24^ In our study, the DCA for DDFS and DFS further validated the predictive consistency and clinical value of the nomograms, respectively, whereas the nomogram predicting OS did not perform as well on DCA as on the other two (Figure 6A–D, Figure S2). However, in general, these three nomogram models, including the STC‐1 expression level, were good indicators of prognosis for patients with BC. In addition, the results of the three prediction models suggested that the prognosis of patients in the high‐risk group was much worse than that of those in the low‐risk group (Figure 7).

## 4 DISCUSSION

STC‐1 is overexpressed in most human cancer tissues, including BC.^10^, ^11^, ^12^, ^13^, ^25^ As a potential therapeutic target of BC, detection of STC‐1 levels in patient blood may be a means of predicting patient prognosis. Numerous BC models have been developed to guide clinical diagnosis and treatment.^26^, ^27^, ^28^ The purpose of this study was to create clinically meaningful nomograms based on STC‐1 for predicting the prognosis of patients with BC.

The STC‐1 content in the peripheral blood of 1653 patients with BC from FDUSCC was detected by ELISA. By analyzing the relationship between STC‐1 expression and clinicopathological features in each patient, we found that the expression of STC‐1 was closely associated with tumor grade, AJCC stage, T stage, N stage, M stage, breast subtype, ER status, PR status, and HER‐2 status. By detecting mRNA levels of STC‐1 in the blood of 58 patients, Wascher et al. found that STC‐1 was associated with primary tumor size, a number of positive lymph nodes, T‐, N‐, and M‐stages, and AJCC stage, which was similar to our results.^29^

Data mining methods can be classified as descriptive and predictive. Among the predictive models include classification and regression.^30^ The essence of regression is to discover the mathematical relationship between the dependent variable and multiple independent variables.^30^ The regression modeling commonly used in clinical research are: multiple linear regression, logistic regression, and Cox regression. Since it is still unclear whether STC‐1 can be an independent prognostic factor for breast cancer, we used COX proportional risk models in this article to predict OS, DFS, and DDFS, respectively.

Alignment diagrams, also known as nomograms, have been widely used in disease diagnosis and clinical practice. Nomograms can integrate multiple risk factors and then plot them together in the same plane using scaled line segments.^31^, ^32^ To prevent model overfitting, the dataset can be divided into a training set for determining the model parameters and a validation set for making a model selection.^33^ The final 1210 patients with BC who met the screening criteria were divided into two datasets at a 7:3 ratio. The KM curves were first plotted to determine indeed that STC‐1 can affect OS, DFS, and DDFS in patients with breast cancer. Based on the results of univariate and multifactorial cox analyses in this study, nomograms of OS, DDFS, and DFS were constructed, and the expression level of STC‐1 was considered an independent risk factor in each model. In addition to STC‐1, negative ER status and N stage were independent prognostic factors for OS, and the risk factors for both DDFS and DFS were T and N stages. Among the various predictive models for BC, the TNM stage of the tumor and hormone receptor status are often considered independent risk factors.^18^, ^28^, ^34^

Good validation of the model is essential for its practical clinical application.^31^, ^32^ The three models predicting OS, DDFS, and DFS showed good accuracy and precision, both in terms of C‐index and AUC values. The calibration curves further validated the consistency of the predicted outcomes of the three models with the actual survival. In addition, DCA examined the clinical utility of the model, predicting that the 3‐ and 5‐year DFS, DDFS, and OS were able to meet the practical needs of clinical decision‐making. Moreover, patients whose STC‐1 levels were in the high‐risk group were much less likely to live than those in the low‐risk group, regardless of the OS, DDFS, or DFS.

The advent of nomograms not only fulfills the need of clinicians for integrated biological and clinical models but also enables the promotion of personalized treatment.^31^, ^35^ In this study, we determined that STC‐1 level in the peripheral blood is closely associated with the prognosis of BC. The development of a BC prediction model using STC‐1 as a biomarker further illustrated the important role of STC‐1 in tumors.

STC‐1 is differentially expressed in BCs of different molecular subtypes; therefore, modeling for a specific type of BC may yield higher efficacy for clinical application, but the sample size collected in our center was not large enough to achieve this goal. Overall, our results demonstrate that STC‐1 can be applied as a critical indicator for predicting OS, DDFS, and DFS of patients with BC. Combining the level of STC‐1 with other recognized independent risk factors to establish a prognostic model may provide new guidance for clinical diagnosis and treatment. Although our models showed good prediction ability under various verification methods, the performance and limitations of the nomogram must be critically reviewed before it is actually applied in the clinic to bring better diagnosis and treatment schemes for patients.

## 5 CONCLUSION

We used STC‐1 levels in the peripheral blood of patients with BC as a liquid biopsy indicator and constructed nomograms with clinicopathological information. Prediction models based on STC‐1 level for prediction of OS, DDFS, and DFS at 3, 5, and 7 years have good predictive ability for BC and have potential clinical value.

## AUTHOR CONTRIBUTIONS

**Sheng Huang:** Conceptualization (equal); data curation (equal); formal analysis (equal); investigation (equal); methodology (equal); project administration (equal); resources (equal); software (equal); supervision (equal); validation (equal); visualization (equal); writing – original draft (equal); writing – review and editing (equal). **Yuyuan Chen:** Conceptualization (equal); data curation (equal); formal analysis (equal); investigation (equal); methodology (equal); project administration (equal); resources (equal); software (equal); supervision (equal); validation (equal); visualization (equal); writing – original draft (equal); writing – review and editing (equal). **Jiong Wu:** Conceptualization (equal); data curation (equal); formal analysis (equal); funding acquisition (equal); investigation (equal); methodology (equal); project administration (equal); resources (equal); software (equal); supervision (equal); validation (equal); visualization (equal); writing – original draft (equal); writing – review and editing (equal). **Yayun Chi:** Conceptualization (equal); data curation (equal); formal analysis (equal); funding acquisition (lead); investigation (equal); methodology (equal); project administration (equal); resources (equal); software (equal); supervision (equal); validation (equal); visualization (equal); writing – original draft (equal); writing – review and editing (lead).

## FUNDING INFORMATION

This work was supported by Yunnan Applied Basic Research Projects‐Union Foundation (202001AY070001‐237 to S.H) and the National Natural Scientific Foundation of China (81772815 to J.W and 81874115 to YY.C).

## CONFLICT OF INTEREST

The authors declare that there are no potential conflicts of interest disclosed.

## RESEARCH INVOLVING HUMAN COMPLIANCE WITH ETHICAL STANDARDS

Approval of the research protocol by an Institutional Reviewer Board: *Yes. (This study was performed in line with the principles of the Declaration of Helsinki. Approval was granted by the* Fudan University Shanghai Cancer Center ethics committee and obtained the informed consent of each patient). *Informed Consent*: N/A. *Registry and the Registration No. of the study/trial*: N/A. *Animal Studies*: N/A.

## Supporting information


Figure S1

Figure S2

Table S1

Table S2

Table S3

Table S4

## Data Availability

All data generated or analyzed during this study are included in this published article.
