# Supplementary figures and images for: Development and validation of novel risk prediction models of breast cancer based on stanniocalcin‐1 level
Source: Cancer Med. 2022 Nov 6;12(6):6499–510. doi: 10.1002/cam4.5419 (PMC10067061; doi:10.1002/cam4.5419)

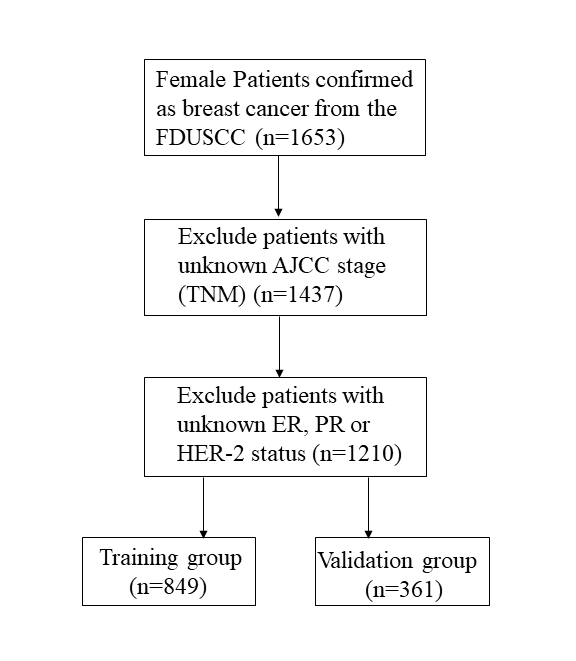

Supplement: Supplementary file 1 — Figure S1 [file CAM4-12-6499-s005.tif]

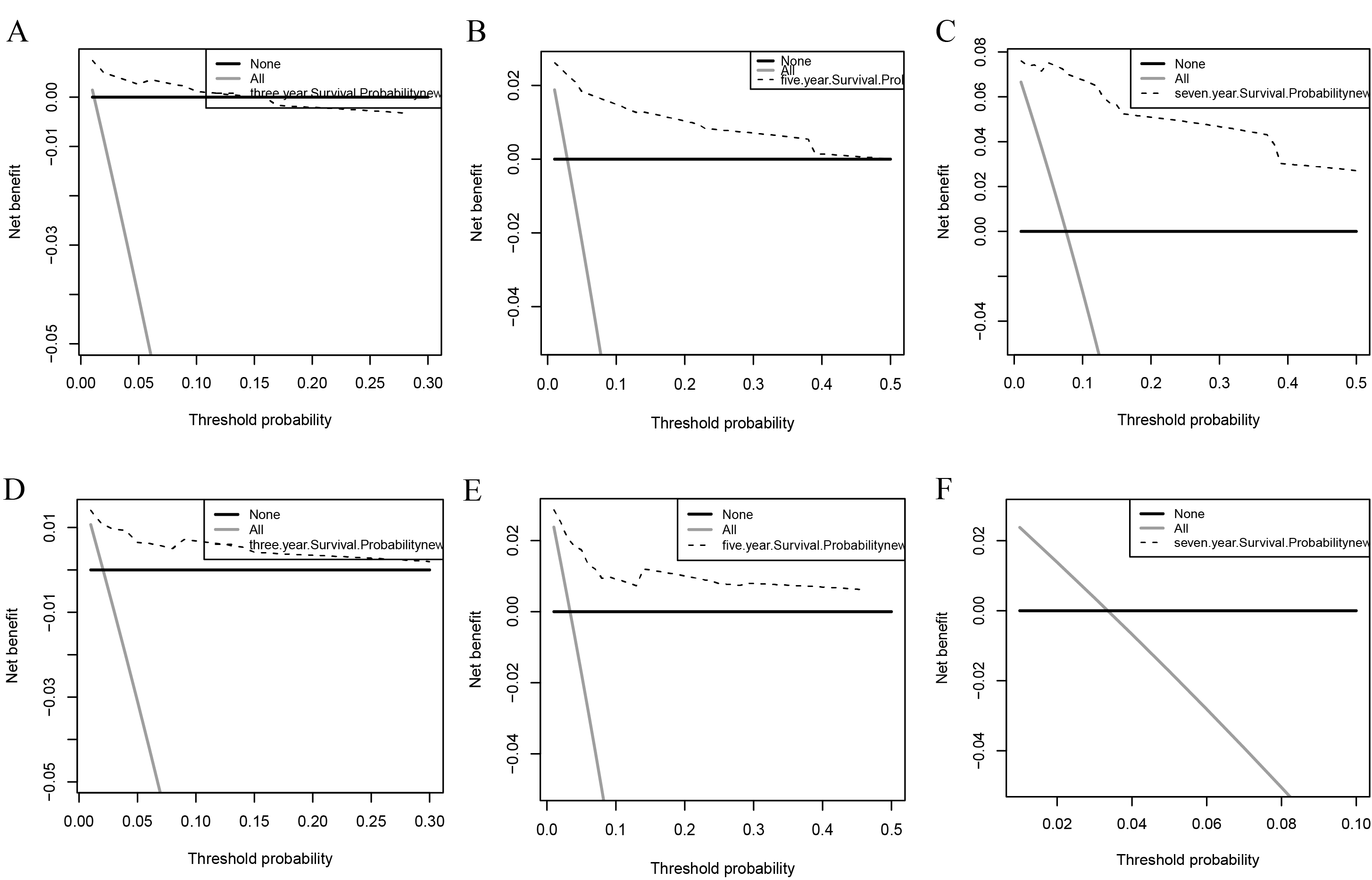

Supplement: Supplementary file 2 — Figure S2 [file CAM4-12-6499-s002.tif]
