# Supplementary material for: Development and validation of novel risk prediction models of breast cancer based on stanniocalcin‐1 level
Source: Cancer Med. 2022 Nov 6;12(6):6499–510. doi: 10.1002/cam4.5419 (PMC10067061; doi:10.1002/cam4.5419)
Supplement: Supplementary file 3 — Table S1 [file CAM4-12-6499-s001.zip › CAM4_5419_Table 1.docx]

| Supplementary Table 1. Characteristics of BC patients. | | | | | | |
| --- | --- | --- | --- | --- | --- | --- |
| Variables | Total cohort | | Training cohort | | Validation cohort | |
|  | N=1210 | | N=849 | | N=361 | |
|  | n | ％ | N | ％ | n | ％ |
| Age |  |  |  |  |  |  |
| ＜51 | 580 | 47.9 | 404 | 47.6 | 176 | 48.8 |
| ≥51 | 630 | 52.1 | 445 | 52.4 | 185 | 51.2 |
| Family history of BC |  |  |  |  |  |  |
| No | 1118 | 92.4 | 784 | 92.3 | 334 | 92.5 |
| Yes | 92 | 7.6 | 65 | 7.7 | 27 | 7.5 |
| Other family history |  |  |  |  |  |  |
| No | 963 | 79.6 | 672 | 79.2 | 291 | 80.6 |
| Yes | 247 | 20.4 | 177 | 20.8 | 70 | 19.4 |
| Stage |  |  |  |  |  |  |
| 0 | 98 | 8.1 | 64 | 7.5 | 34 | 9.4 |
| I | 348 | 28.8 | 242 | 28.5 | 106 | 29.4 |
| II | 541 | 44.7 | 385 | 45.3 | 156 | 43.0 |
| III | 223 | 18.4 | 158 | 18.6 | 65 | 18.0 |
| T |  |  |  |  |  |  |
| T0 | 106 | 8.8 | 67 | 7.9 | 39 | 10.8 |
| T1 | 560 | 46.3 | 393 | 46.3 | 167 | 46.3 |
| T2 | 503 | 41.6 | 366 | 43.1 | 137 | 38.0 |
| T3 | 41 | 3.4 | 23 | 2.7 | 18 | 5.0 |
| N |  |  |  |  |  |  |
| N0 | 704 | 58.2 | 494 | 58.2 | 210 | 58.2 |
| N1 | 290 | 24.0 | 201 | 23.7 | 89 | 24.7 |
| N2 | 114 | 9.4 | 86 | 10.1 | 28 | 7.8 |
| N3 | 102 | 8.4 | 68 | 8.0 | 34 | 9.4 |
| Grade |  |  |  |  |  |  |
| I | 22 | 1.8 | 13 | 1.5 | 9 | 2.5 |
| II | 639 | 52.8 | 448 | 52.8 | 191 | 52.9 |
| III | 326 | 26.9 | 233 | 27.4 | 93 | 25.8 |
| IV | 223 | 18.4 | 155 | 18.3 | 68 | 18.8 |
| Breast subtype |  |  |  |  |  |  |
| HR+/HER2- (Luminal A) | 899 | 74.3 | 625 | 73.6 | 274 | 75.9 |
| HR+/HER2+ (Luminal B) | 57 | 4.7 | 41 | 4.8 | 16 | 4.4 |
| HR-/HER2+ (HER2 enriched) | 28 | 2.3 | 21 | 2.5 | 7 | 1.9 |
| HR-/HER2- (Triple Negative) | 226 | 18.7 | 162 | 19.1 | 64 | 17.7 |
| ER |  |  |  |  |  |  |
| Negative | 307 | 25.4 | 223 | 26.3 | 84 | 23.3 |
| Positive | 903 | 74.6 | 626 | 73.7 | 277 | 76.7 |
| PR |  |  |  |  |  |  |
| Negative | 371 | 30.7 | 261 | 30.7 | 110 | 30.5 |
| Positive | 839 | 69.3 | 588 | 69.3 | 251 | 69.5 |
| HER-2 |  |  |  |  |  |  |
| Negative | 1125 | 93.0 | 787 | 92.7 | 338 | 93.6 |
| Positive | 85 | 7.0 | 62 | 7.3 | 23 | 6.4 |
| Ki67 |  |  |  |  |  |  |
| Negative | 206 | 17.0 | 151 | 17.8 | 55 | 15.2 |
| Positive | 192 | 15.9 | 125 | 14.7 | 67 | 18.6 |
| Unknown | 812 | 67.1 | 573 | 67.5 | 239 | 66.2 |
| Histological type |  |  |  |  |  |  |
| 0 | 112 | 9.3 | 69 | 8.1 | 43 | 11.9 |
| 1 | 995 | 82.2 | 703 | 82.8 | 292 | 80.9 |
| 2 | 12 | 1.0 | 9 | 1.1 | 3 | 0.8 |
| 3 | 46 | 3.8 | 32 | 3.8 | 14 | 3.9 |
| Unknown | 45 | 3.7 | 36 | 4.2 | 9 | 2.5 |
| Vascular tumor emboli |  |  |  |  |  |  |
| Negative | 648 | 53.6 | 460 | 54.2 | 188 | 52.1 |
| Positive | 324 | 26.8 | 222 | 26.1 | 102 | 28.3 |
| Unknown | 238 | 19.7 | 167 | 19.7 | 71 | 19.7 |
| STC-1 |  |  |  |  |  |  |
| 0-0.3 μg/ml | 877 | 72.5 | 617 | 72.7 | 260 | 72.0 |
| >0.3 μg/ml | 333 | 27.5 | 232 | 27.3 | 101 | 28.0 |
