# Supplementary material for: Development and validation of novel risk prediction models of breast cancer based on stanniocalcin‐1 level
Source: Cancer Med. 2022 Nov 6;12(6):6499–510. doi: 10.1002/cam4.5419 (PMC10067061; doi:10.1002/cam4.5419)
Supplement: Supplementary file 3 — Table S1 [file CAM4-12-6499-s001.zip › cam45419-sup-0003-TableS1.docx]

| Supplementary Table 1. Univariate and multifactorial Cox analyses of OS in BC. | | | | | | |
| --- | --- | --- | --- | --- | --- | --- |
| Characteristics |  | Univariate analysis | |  | Multivariate analysis | |
|  |  | HR (95% CI) | P |  | HR (95% CI) | P |
| Age |  |  |  |  |  |  |
| ＜51 |  | Reference |  |  |  |  |
| ≥51 |  | 0.693(0.318-1.509) | 0.355 |  |  |  |
| Family history of BC |  |  |  |  |  |  |
| No |  | Reference |  |  |  |  |
| Yes |  | 1.814(0.625-5.271) | 0.274 |  |  |  |
| Other family history |  |  |  |  |  |  |
| No |  | Reference |  |  |  |  |
| Yes |  | 1.42(0.595-3.39) | 0.430 |  |  |  |
| Stage |  |  |  |  |  |  |
| 0 |  | Reference | <0.001 |  |  |  |
| I |  | 1223.165(0-2.277E+68) | 0.926 |  |  |  |
| II |  | 6386.193(0-1.175E+69) | 0.909 |  |  |  |
| III |  | 46983.046(0-8.642E+69) | 0.888 |  |  |  |
| T |  |  |  |  |  |  |
| T0 |  | Reference | 0.009^*^ |  | Reference | 0.465 |
| T1 |  | 5248.866(1.212E-68-2.272E+75) | 0.919 |  | 6659.052(1.785E-90-2.484E+97) | 0.936 |
| T2 |  | 24357.079(5.635E-68-1.052E+76) | 0.904 |  | 15179.271(4.071E-90-5.660E+97) | 0.930 |
| T3 |  | 53729.795(1.237E-67-2.334E+76) | 0.897 |  | 10885.182(2.905E-90-4.079E+97) | 0.933 |
| N |  |  |  |  |  |  |
| N0 |  | Reference | <0.001^*^ |  | Reference | 0.001^*^ |
| N1 |  | 2.416(0.603-9.672) | 0.213 |  | 2.177(0.481-9.316) | 0.321 |
| N2 |  | 11.619(3.262-41.387) | <0.001 |  | 7.387(1.894-28.810) | 0.004 |
| N3 |  | 24.845(8.009-77.076) | <0.001 |  | 12.592(3.354-47.277) | <0.001 |
| Grade |  |  |  |  |  |  |
| I |  | Reference | 0.069 |  |  |  |
| II |  | 1810.716(3.784E-81-8.664E+86) | 0.939 |  |  |  |
| III |  | 6233.399(1.303E-80-2.981E+87) | 0.929 |  |  |  |
| IV |  | 5145.737(1.076E-80-2.462E+87) | 0.931 |  |  |  |
| Breast subtype |  |  |  |  |  |  |
| Luminal A |  | Reference | 0.822 |  |  |  |
| Luminal B |  | 6.260E-06(0-) | 0.983 |  |  |  |
| HER-2 enriched |  | 1.96(0.261-14.725) | 0.513 |  |  |  |
| Triple Negative |  | 1.411(0.589-3.38) | 0.440 |  |  |  |
| ER |  |  |  |  |  |  |
| Negative |  | Reference |  |  | Reference |  |
| Positive |  | 0.354(0.164-0.763) | 0.008^*^ |  | 0.287(0.120-0.689) | 0.005^*^ |
| PR |  |  |  |  |  |  |
| Negative |  | Reference |  |  |  |  |
| Positive |  | 0.590(0.273-1.277) | 0.181 |  |  |  |
| HER-2 |  |  |  |  |  |  |
| Negative |  | Reference |  |  |  |  |
| Positive |  | 0.899(0.120-6.711) | 0.917 |  |  |  |
| Ki67 |  |  |  |  |  |  |
| Negative |  | Reference | 0.685 |  |  |  |
| Positive |  | 1.484(0.332-6.644) | 0.605 |  |  |  |
| Unknown |  | 0.913(0.263-3.161) | 0.885 |  |  |  |
| Histological type |  |  |  |  |  |  |
| DCIS |  | Reference | 1 |  |  |  |
| IDC |  | 74685.75(1.300E-151-4.293E+160) | 0.951 |  |  |  |
| ILC |  | 1.003(0-) | 1.000 |  |  |  |
| MBC |  | 0.999(1.726E-261-5.785E+260) | 1.000 |  |  |  |
| Unknown |  | 87517.682(1.514E-151-5.057E+160) | 0.950 |  |  |  |
| Vascular tumor emboli |  |  |  |  |  |  |
| Negative |  | Reference | 0.009^*^ |  | Reference | 0.880 |
| Positive |  | 3.391(1.421-8.09) | 0.006 |  | 1.040(0.390-2.769) | 0.938 |
| Unknown |  | 1.043(0.311-3.499) | 0.946 |  | 1.395(0.374-5.205) | 0.621 |
| STC-1 |  |  |  |  |  |  |
| 0-0.3 μg/ml |  | Reference |  |  | Reference |  |
| >0.3 μg/ml |  | 27.174(7.997-92.343) | <0.001^*^ |  | 19.123(5.530-66.127) | <0.001^*^ |
| ^*^*P*<0.05 |  |  |  |  |  |  |
