# Supplementary material for: Development and validation of novel risk prediction models of breast cancer based on stanniocalcin‐1 level
Source: Cancer Med. 2022 Nov 6;12(6):6499–510. doi: 10.1002/cam4.5419 (PMC10067061; doi:10.1002/cam4.5419)
Supplement: Supplementary file 4 — Table S2 [file CAM4-12-6499-s004.docx]

| Supplementary Table 2. Univariate and multifactorial Cox analysis based on DDFS of BC. | | | | | | |
| --- | --- | --- | --- | --- | --- | --- |
| Characteristics |  | Univariate analysis | |  | Multivariate analysis | |
|  |  | HR (95% CI) | P |  | HR (95% CI) | P |
| Age |  |  |  |  |  |  |
| ＜51 |  | Reference |  |  |  |  |
| ≥51 |  | 1.104(0.728-1.676) | 0.641 |  |  |  |
| Family history of BC |  |  |  |  |  |  |
| No |  | Reference |  |  |  |  |
| Yes |  | 0.812(0.355-1.86) | 0.623 |  |  |  |
| Other family history |  |  |  |  |  |  |
| No |  | Reference |  |  |  |  |
| Yes |  | 1.333(0.835-2.129) | 0.229 |  |  |  |
| Unknown |  |  |  |  |  |  |
| Stage |  |  |  |  |  |  |
| 0 |  | Reference | <0.001^*^ |  |  |  |
| I |  | 0.778(0.162-3.746) | 0.754 |  |  |  |
| II |  | 3.201(0.775-13.216) | 0.108 |  |  |  |
| III |  | 7.997(1.927-33.182) | 0.004 |  |  |  |
| T |  |  |  |  |  |  |
| T0 |  | Reference | <0.001^*^ |  | Reference | 0.014^*^ |
| T1 |  | 1.290(0.391-4.264) | 0.676 |  | 0.714(0.204-2.504) | 0.599 |
| T2 |  | 3.061(0.957-9.797) | 0.059 |  | 1.363(0.392-4.733) | 0.626 |
| T3 |  | 7.665(1.982-29.649) | 0.003 |  | 2.482(0.589-10.457) | 0.215 |
| N |  |  |  |  |  |  |
| N0 |  | Reference | <0.001^*^ |  | Reference | <0.001^*^ |
| N1 |  | 4.095(2.350-7.137) | <0.001 |  | 4.236(2.322-7.727) | <0.001 |
| N2 |  | 5.499(2.849-10.616) | <0.001 |  | 4.782(2.369-9.652) | <0.001 |
| N3 |  | 9.246(4.973-17.191) | <0.001 |  | 7.362(3.600-15.053) | <0.001 |
| Grade |  |  |  |  |  |  |
| I |  | Reference | 0.064 |  |  |  |
| II |  | 2660.342(1.178E-39-6.005E+45) | 0.874 |  |  |  |
| III |  | 4736.85(2.098E-39-1.0694E+46) | 0.865 |  |  |  |
| IV |  | 2591.148(1.147E-39-5.855E+45) | 0.874 |  |  |  |
| Breast subtype |  |  |  |  |  |  |
| Luminal A |  | Reference | 0.243 |  |  |  |
| Luminal B |  | 1.215(0.442-3.341) | 0.706 |  |  |  |
| HER2 enriched |  | 2.324(0.936-5.771) | 0.069 |  |  |  |
| Triple Negative |  | 0.823(0.462-1.465) | 0.508 |  |  |  |
| ER |  |  |  |  |  |  |
| Negative |  | Reference |  |  |  |  |
| Positive |  | 0.867(0.546-1.376) | 0.545 |  |  |  |
| PR |  |  |  |  |  |  |
| Negative |  | Reference |  |  |  |  |
| Positive |  | 1.045(0.665-1.642) | 0.850 |  |  |  |
| HER-2 |  |  |  |  |  |  |
| Negative |  | Reference |  |  |  |  |
| Positive |  | 1.717(0.860-3.426) | 0.125 |  |  |  |
| Ki67 |  |  |  |  |  |  |
| Negative |  | Reference | 0.328 |  |  |  |
| Positive |  | 1.093(0.546-2.190) | 0.801 |  |  |  |
| Unknown |  | 0.751(0.436-1.296) | 0.304 |  |  |  |
| Histological type |  |  |  |  |  |  |
| DCIS |  | Reference | 0.154 |  |  |  |
| IDC |  | 2.429(0.768-7.689) | 0.131 |  |  |  |
| ILC |  | 4.426(0.739-26.494) | 0.103 |  |  |  |
| MBC |  | 0.617(0.064-5.929) | 0.675 |  |  |  |
| Unknown |  | 0.610(0.063-5.869) | 0.669 |  |  |  |
| Vascular tumor emboli |  |  |  |  |  |  |
| Negative |  | Reference | <0.001^*^ |  | Reference | 0.802 |
| Positive |  | 2.399(1.519-3.79) | 0.000 |  | 0.938(0.567-1.551) | 0.802 |
| Unknown |  | 1.21(0.658-2.224) | 0.539 |  | 1.167(0.624-2.181) | 0.629 |
| STC-1 |  |  |  |  |  |  |
| <0.3 μg/ml |  | Reference |  |  | Reference |  |
| 0-0.3 μg/ml |  | 1.915(1.248-2.937) | 0.003^*^ |  | 1.796(1.161-2.777) | 0.008^*^ |
| ^*^*P*<0.05 |  |  |  |  |  |  |
