# Supplementary material for: Development and validation of novel risk prediction models of breast cancer based on stanniocalcin‐1 level
Source: Cancer Med. 2022 Nov 6;12(6):6499–510. doi: 10.1002/cam4.5419 (PMC10067061; doi:10.1002/cam4.5419)
Supplement: Supplementary file 5 — Table S3 [file CAM4-12-6499-s006.docx]

Supplementary Table 4. C-index of prediction models.

| Nomogram | C-index(95%CI) | |
| --- | --- | --- |
|  | Training cohort | Validation cohort |
| OS | 0.888(0.781-0.994) | 0.807(0.642-0.972) |
| DDFS | 0.746(0.694-0.798) | 0.697(0.600-0.794) |
| DFS | 0.711(0.659-0.764) | 0.705(0.618-0.792) |
