# Supplementary material for: Development and validation of novel risk prediction models of breast cancer based on stanniocalcin‐1 level
Source: Cancer Med. 2022 Nov 6;12(6):6499–510. doi: 10.1002/cam4.5419 (PMC10067061; doi:10.1002/cam4.5419)
Supplement: Supplementary file 6 — Table S4 [file CAM4-12-6499-s003.docx]

| Supplementary Table 3. Univariate and multifactorial Cox analysis based on DFS of BC. | | | | | | |
| --- | --- | --- | --- | --- | --- | --- |
| Characteristics |  | Univariate analysis | |  | Multivariate analysis | |
|  |  | HR (95% CI) | P |  | HR (95% CI) | P |
| Age |  |  |  |  |  |  |
| ＜51 |  | Reference |  |  |  |  |
| ≥51 |  | 1.149(0.793-1.665) | 0.462 |  |  |  |
| Family history of BC |  |  |  |  |  |  |
| No |  | Reference |  |  |  |  |
| Yes |  | 0.840(0.409-1.724) | 0.634 |  |  |  |
| Other family history |  |  |  |  |  |  |
| No |  | Reference |  |  |  |  |
| Yes |  | 1.191(0.777-1.826) | 0.422 |  |  |  |
| Stage |  |  |  |  |  |  |
| 0 |  | Reference | <0.001^*^ |  |  |  |
| I |  | 1.801(0.414-7.832) | 0.433 |  |  |  |
| II |  | 3.536(0.859-14.561) | 0.080 |  |  |  |
| III |  | 10.481(2.547-43.124) | 0.001 |  |  |  |
| T |  |  |  |  |  |  |
| T0 |  | Reference | <0.001^*^ |  | Reference | 0.088 |
| T1 |  | 1.436(0.513-4.025) | 0.491 |  | 0.949(0.324-2.776) | 0.923 |
| T2 |  | 2.804(1.021-7.702) | 0.045 |  | 1.473(0.503-4.313 | 0.479 |
| T3 |  | 5.984(1.751-20.45) | 0.004 |  | 2.274(0.624-8.296) | 0.213 |
| N |  |  |  |  |  |  |
| N0 |  | Reference | <0.001^*^ |  | Reference | <0.001^*^ |
| N1 |  | 2.772(1.709-4.496) | <0.001 |  | 2.901(1.727-4.873) | <0.001 |
| N2 |  | 4.875(2.822-8.423) | <0.001 |  | 4.484(2.500-8.043) | <0.001 |
| N3 |  | 7.330(4.326-12.421) | <0.001 |  | 6.467(3.504-11.935) | <0.001 |
| Grade |  |  |  |  |  |  |
| I |  | Reference | 0.192 |  |  |  |
| II |  | 2741.428(3.604E-34-2.0854E+40) | 0.855 |  |  |  |
| III |  | 4200.775(5.522E-34-3.195E+40) | 0.847 |  |  |  |
| Unknown |  | 2738.803(3.597E-34-2.085E+40) | 0.855 |  |  |  |
| Breast subtype |  |  |  |  |  |  |
| Luminal A |  | Reference | 0.511 |  |  |  |
| Luminal B |  | 0.952(0.348-2.600) | 0.923 |  |  |  |
| HER2 enriched |  | 1.865(0.756-4.600) | 0.176 |  |  |  |
| Triple Negative |  | 0.871(0.530-1.433) | 0.588 |  |  |  |
| ER |  |  |  |  |  |  |
| Negative |  | Reference |  |  |  |  |
| Positive |  | 0.890(0.589-1.345) | 0.580 |  |  |  |
| PR |  |  |  |  |  |  |
| Negative |  | Reference |  |  |  |  |
| Positive |  | 1.109(0.739-1.664) | 0.617 |  |  |  |
| HER-2 |  |  |  |  |  |  |
| Negative |  | Reference |  |  |  |  |
| Positive |  | 1.343(0.678-2.66) | 0.397 |  |  |  |
| Ki67 |  |  |  |  |  |  |
| Negative |  | Reference | 0.113 |  |  |  |
| Positive |  | 1.321(0.716-2.438) | 0.373 |  |  |  |
| Unknown |  | 0.793(0.482-1.307) | 0.364 |  |  |  |
| Histological type |  |  |  |  |  |  |
| DCIS |  | Reference | 0.194 |  |  |  |
| IDC |  | 1.858(0.757-4.559) | 0.176 |  |  |  |
| ILC |  | 2.643(0.512-13.629) | 0.246 |  |  |  |
| MBC |  | 0.374(0.044-3.201) | 0.369 |  |  |  |
| Unknown |  | 0.753(0.146-3.886) | 0.735 |  |  |  |
| Vascular tumor emboli |  |  |  |  |  |  |
| Negative |  | Reference | 0.002^*^ |  | Reference | 0.628 |
| Positive |  | 2.048(1.364-3.077) | 0.001 |  | 0.885(0.564-1.391) | 0.597 |
| Unknown |  | 1.163(0.687-1.969) | 0.573 |  | 1.172(0.683-2.011) | 0.564 |
| STC-1 |  |  |  |  |  |  |
| <0.3 μg/ml |  | Reference |  |  | Reference |  |
| >0.3 μg/ml |  | 1.858(1.268-2.724) | 0.001^*^ |  | 1.712(1.161-2.525) | 0.007^*^ |
| ^*^*P*<0.05 |  |  |  |  |  |  |
